# Association of Physical Activity Level With Risk of Dementia in a Nationwide Cohort in Korea

**DOI:** 10.1001/jamanetworkopen.2021.38526

**Published:** 2021-12-16

**Authors:** Minjae Yoon, Pil-Sung Yang, Moo-Nyun Jin, Hee Tae Yu, Tae-Hoon Kim, Eunsun Jang, Jae-Sun Uhm, Hui-Nam Pak, Moon-Hyoung Lee, Boyoung Joung

**Affiliations:** 1 Division of Cardiology, Department of Internal Medicine, Severance Cardiovascular Hospital, Yonsei University College of Medicine, Seoul, Republic of Korea; 2 Division of Cardiology, CHA Bundang Medical Center, CHA University, Seongnam, Republic of Korea; 3 Division of Cardiology, Department of Internal Medicine, Sangye Paik Hospital, Inje University College of Medicine, Seoul, Republic of Korea

## Abstract

**Importance:**

Current guidelines recommend 500 to 999 metabolic equivalent (MET)–minutes per week (MET-min/wk) of regular physical activity. However, evidence regarding the association between light-intensity physical activity (LPA) and dementia in older adults is inconsistent.

**Objective:**

To assess the association between physical activity and new-onset dementia, focusing on the dose-response association between physical activity and dementia and the association of LPA with the incidence of dementia.

**Design, Setting, and Participants:**

For this nationwide retrospective cohort study, we analyzed 62 286 participants aged 65 years or older without preexisting dementia who had available health checkup data from the Korean National Health Insurance Service database from January 2009 to December 2012. Participants were followed up until December 31, 2013, and data analysis was performed from July 2020 to January 2021.

**Exposures:**

Physical activity level was assessed using a standardized, self-reported questionnaire at baseline. Physical activity–related energy expenditure (in MET-min/wk) was calculated by summing the product of frequency, intensity, and duration.

**Main Outcomes and Measures:**

Incidence of dementia. Incidence rates were calculated by dividing the number of events by the person-time at risk (presented as the incidence per 1000 person-years). Hazard ratios (HRs) and 95% CIs for dementia were analyzed according to physical activity level. Competing risk regression was performed by using the Fine-Gray subdistribution hazard model, with mortality as the competing risk for dementia events. Multivariable regression models were constructed with adjustment for various patient characteristics. Incident dementia occurring 2 years after enrollment was assessed, and separate analyses included all follow-up periods. Restricted cubic spline curves were used to examine the association of continuous values of physical activity with dementia.

**Results:**

Among 62 286 participants, 60.4% were women, and the mean (SD) age was 73.2 (5.3) years. During a median follow-up of 42 months, 3757 participants (6.0%) developed dementia, and the overall incidence was 21.6 per 1000 person-years. Compared with inactive individuals (0 MET-min/wk), insufficiently active (1-499 MET-min/wk; mean, 284 MET-min/wk), active (500-999 MET-min/wk; mean, 675 MET-min/wk), and highly active participants (≥1000 MET-min/wk; mean, 1627 MET-min/wk) showed 10% (adjusted hazard ratio [HR], 0.90; 95% CI, 0.81-0.99), 20% (adjusted HR, 0.80; 95% CI, 0.71-0.92), and 28% (adjusted HR, 0.72; 95% CI, 0.60-0.83) reduced dementia risk, respectively. Thus, a progressive decrease in the adjusted HR of dementia was associated with increasing physical activity level, and a restricted cubic spline curve showed that this association started with a low amount of total physical activity. This association was consistent regardless of age, sex, and other comorbidities or after censoring for stroke. Compared with total sedentary behavior, even a low amount of LPA (1-299 MET-min/wk; mean, 189 MET-min/wk) was associated with reduced dementia risk (adjusted HR, 0.86; 95% CI, 0.74-0.99).

**Conclusions and Relevance:**

In older adults, an increased physical activity level, including a low amount of LPA, was associated with a reduced risk of dementia. Promotion of LPA might reduce the risk of dementia in older adults.

## Key Points

**Question:**

Is physical activity, especially light-intensity physical activity, independently associated with new-onset dementia?

**Findings:**

In this nationwide cohort study that included 62 286 participants in Korea, an increased physical activity level, including a low amount of light-intensity activity, was associated with a reduced risk of dementia in older adults.

**Meaning:**

This study’s findings suggest that an increased level of physical activity in older adults, including a low amount of light-intensity physical activity, may be associated with a reduction in the risk of dementia.

## Teaser

This cohort study uses data from the Korean National Health Insurance Service database to assess the association between physical activity and new-onset dementia with a focus on the dose-response association between physical activity and the incidence of dementia.

## Introduction

Physical activity has been shown to be associated with a reduced risk of vascular and nonvascular diseases as well as mortality.^1^ Current guidelines^2,3^ recommend at least 150 to 300 minutes of moderate-intensity physical activity (MPA) or 75 to 150 minutes of vigorous-intensity physical activity (VPA) per week, which is equivalent to 500 to 999 metabolic equivalent (MET)–minutes per week (MET-min/wk) in older adults (aged ≥65 years). Performing more than 150 minutes of moderate-intensity aerobic exercise (eg, brisk walking) per week is associated with at least 30% lower risk of morbidity, mortality, disability, and frailty compared with being inactive.^4,5,6^ Moreover, some studies^7,8^ reported that the effect of light-intensity physical activity (LPA) might be beneficial for older adults. Thus, current guidelines for older adults suggest replacing sedentary time with physical activity of any intensity, including LPA.^3^

Several studies^9,10,11,12,13^ have shown that physical activity is also associated with a reduced risk of dementia, and the current World Health Organization guidelines on dementia^14^ recommend that adults with normal cognition should perform physical activities to reduce the risk of cognitive decline. However, some studies^15,16,17,18,19^ have reported that physical activity might not be associated with a reduced risk of dementia, suggesting that previous findings showing a lower risk of dementia in physically active people could be attributed to reverse causation. Inconsistent research findings on exercise intensity were also reported,^8,20^ particularly in relation to whether LPA was associated with a reduced risk of dementia. Many older adults cannot perform physical activities beyond MPA because of their frailty and comorbidities; therefore, these adults would have to gain the benefits of physical activity from LPA.

To help clarify this association, we analyzed the association of physical activity with new-onset dementia in older adults in a nationwide Korean cohort. We focused on the dose-response association between physical activity and dementia and explored the association of LPA with incidence of dementia.

## Methods

### Study Population

This was a nationwide retrospective cohort study. Data were collected from the National Health Insurance Service (NHIS)–Senior database of Korea, comprising 558 147 individuals who were recruited using the 10% simple random sampling method from a total of 5.5 million adults aged 60 years or older in the National Health Information Database.^21,22^ The NHIS-Senior database of Korea comprised the following data: sociodemographic and socioeconomic information, health checkup examinations, insurance status, and medical history records. This study was approved by the institutional review board of the Yonsei University Health System. The requirement for informed consent was waived because personal identifying information was removed after cohort creation according to strict confidentiality guidelines. This study followed the Strengthening the Reporting of Observational Studies in Epidemiology (STROBE) reporting guideline.^23^

Between 2009 and 2012, this study enrolled 68 216 participants 65 years or older with available health checkup data from the Korean NHIS-Senior database. We excluded participants with a history of any type of dementia before cohort enrollment (n = 5930). Therefore, a total of 62 286 individuals were enrolled and followed up until December 2013. Therefore, a total of 62286 individuals were enrolled and followed up until December 2013. Data analysis was performed from July 2020 to January 2021.

### Physical Activity Level Assessment

The leisure-time physical activity level was assessed with self-report–structured questionnaires using a 7-day recall method (eFigure 1 in the Supplement).^24^ The survey included 3 questions regarding the usual frequency (days per week) of (1) VPA for at least 20 minutes, (2) MPA for at least 30 minutes, and (3) LPA for at least 30 minutes. Vigorous-intensity physical activity was defined as intense exercise that caused severe shortness of breath, including running and bicycling at high speed; MPA, as exercise that caused mild shortness of breath, including brisk walking and bicycling at usual speed; and LPA, as walking at a slow or leisurely pace.

Based on findings from previous studies,^3,25,26,27^ ratings of 3.0, 4.0, and 8.0 METs were assigned for LPA, MPA, and VPA, respectively. Physical activity–related energy expenditure was calculated in METs in minutes per week by summing the product of frequency, intensity, and duration. In consideration of the current guidelines and findings of previous studies,^2,3,26,28^ participants were stratified on the basis of their weekly total physical activity levels as follows: (1) inactive group, no leisure-time physical activity beyond basic movements; (2) insufficiently active group, less than the recommended target range (1-499 MET-min/wk); (3) active group, meeting the recommended target range (500-999 MET-min/wk); and (4) highly active group, exceeding the recommended target range (≥1000 MET-min/wk).

### Baseline Comorbidities

Baseline comorbidities were identified from the *International Statistical Classification of Diseases and Related Health Problems, Tenth Revision* (*ICD-10*) codes and prescription medications prior to health checkups (eTable 1 in the Supplement). To ensure diagnostic accuracy, participants were considered to have comorbidities when the condition was a discharge diagnosis or was confirmed at least twice in an outpatient setting, similar to criteria used in previous studies.^29,30,31^ The Hospital Frailty Risk score was calculated retrospectively using 109 *ICD-10* diagnostic codes that have been found to be associated with frailty (eTable 2 in the Supplement).^32^

### Assessment of Dementia

The study end point was the initial occurrence of dementia. Secondary outcomes were the development of dementia subtypes, including Alzheimer disease (AD) and vascular dementia. A diagnosis of dementia was based on the *ICD-10* codes for dementia (F00 or G30 for AD; F01 for vascular dementia; and F02, F03, or G31 for other types of dementia) and prescription of 1 or more medications for dementia (rivastigmine, galantamine, memantine, or donepezil).^31,33^ To evaluate the accuracy of this definition, validation studies were performed by internal medicine physicians in our study in 2 hospitals for 972 participants by using the patients’ medical records and cognitive function test results, resulting in a positive predictive value of 94.7%.^22,34^ Cognitive function at baseline was examined using a simple prescreening tool, the Korean Dementia Screening Questionnaire (KDSQ), which is used to prescreen individuals for dementia in routine Korean national health checkups; it was administered to some of the study participants (9496 [15.2%]) who underwent additional screening during a life transition period when major changes in health or lifestyle occurred. The details of KDSQ are presented in the eMethods in the Supplement.

### Statistical Analysis

Descriptive statistics were used to characterize baseline characteristics and comorbidities. Categorical variables were reported as frequencies (percentages), and continuous variables were expressed as means and SDs. The categorical variables were compared using the Fisher exact test or the Pearson χ^2^ test, and continuous variables were compared using the *t* test. An absolute standardized difference of less than 0.1 (10%) was regarded as a negligible difference between the study groups.

Incidence rates of dementia were calculated by dividing the number of events by the person-time at risk and are presented as the incidence per 1000 person-years. We analyzed the hazard ratios (HRs) and 95% CIs for dementia according to the physical activity level. Competing risk regression was performed by using the Fine-Gray subdistribution hazard model,^35^ wherein mortality was set as the competing risk for dementia events. Multivariable regression models were constructed with adjustment for age, sex, body mass index, Hospital Frailty Risk score, annual income, smoking, alcohol, hypertension, diabetes, dyslipidemia, chronic kidney disease, heart failure, vascular disease, prior ischemic stroke or transient ischemic attack, chronic obstructive pulmonary disease, and malignancy. We conducted main analyses by excluding the first 2 years of follow-up to minimize reverse causation bias. Incident dementia occurring 2 years after enrollment was then assessed. We also performed separate analyses by including all follow-up periods. We used restricted cubic spline curves to examine the association of continuous values of physical activity (0 MET-min/wk as reference) with dementia. In addition, we assessed the association of LPA with dementia in participants who did not perform any activity beyond MPA. The LPA level was categorized as no LPA (total sedentary behavior), 1 to 299 MET-min/wk of LPA, or 300 or more MET-min/wk of LPA.

We conducted subgroup analyses for the primary outcome stratified by age, sex, and baseline comorbidities. Interaction tests were performed for all subgroups. We additionally performed separate analyses by censoring participants at the date of stroke if the stroke occurred before the end of the follow-up period.

All tests were 2-tailed, and *P* < .05 was considered significant. Statistical analyses were conducted using R, version 4.0.2 (R Foundation for Statistical Computing).

We performed sensitivity analyses by first excluding patients with a KDSQ score of 4 or greater (n = 2220), who were likely experiencing dementia at baseline, to reduce the possibility of including participants with dementia. Second, we performed a time-varying regression, wherein physical activity levels were treated as a time-dependent variable among participants who completed follow-up questionnaires after their baseline visit.

## Results

### Participant Characteristics at Baseline

In total, 62 286 participants (male, 39.6%; female, 60.4%) with a mean (SD) age of 73.2 (5.3) years were included in this analysis. Participants’ demographic data, comorbidities, and laboratory findings according to leisure-time physical activity level are summarized in the Table. In this study, 35.0% of participants belonged to the inactive group; 25.0%, to the insufficiently active group (mean, 284 MET-min/wk); 24.4%, to the active group (mean, 675 MET-min/wk); and 15.2%, to the highly active group (mean, 1627 MET-min/wk).

**Table.** Baseline Characteristics of Participants Stratified by Leisure-Time Physical Activity Level

Characteristic	No. (%)					ASD, %
	All (N = 62 286)	Physical activity level (MET-min/wk)				
		Inactive (0) [n = 21 826]	Insufficiently active (1-499) [n = 15 562]	Active (500-999) [n = 15 346]	Highly active (≥1000) [n = 9552]	
Age, mean (SD), y	73.2 (5.3)	74.1 (5.9)	73.2 (5.3)	72.6 (4.8)	71.8 (4.3)	24.1
Age groups, y						
65-74	42 712 (68.6)	13 531 (62.0)	10 538 (67.7)	11 114 (72.4)	7529 (78.8)	22.1
75-84	17 135 (27.5)	6938 (31.8)	4441 (28.5)	3879 (25.3)	1877 (19.7)	
≥85	2439 (3.9)	1357 (6.2)	583 (3.7)	353 (2.3)	146 (1.5)	
Women	37 598 (60.4)	14 509 (66.5)	10 149 (65.2)	8747 (57.0)	4193 (43.9)	
Men	24 688 (39.6)	7317 (33.5)	5413 (34.8)	6599 (43.0)	5359 (56.1)	26.0
BMI, mean (SD), kg/m^2^	23.9 (3.4)	23.8 (3.6)	24.0 (3.4)	23.9 (3.2)	24.0 (3.0)	3.7
Waist circumference, mean (SD), cm	83.2 (8.8)	83.0 (9.2)	83.3 (8.8)	83.3 (8.5)	83.8 (8.4)	4.8
Blood pressure, mean (SD), mm Hg						
Systolic	131.6 (17.0)	131.9 (17.5)	131.4 (16.8)	131.4 (16.9)	131.6 (16.5)	1.9
Diastolic	78.4 (10.4)	78.5 (10.7)	78.4 (10.4)	78.2 (10.2)	78.2 (10.3)	2.0
Hospital Frailty Risk score, median (IQR)	0 (0-1.8)	0 (0-2.3)	0 (0-1.8)	0 (0-1.4)	0 (0-1.2)	12.2
High tertile of annual income^a^	29 417 (47.2)	9677 (44.3)	7496 (48.2)	7366 (48.0)	4878 (51.1)	6.8
Smoker (ex-smoker or current)	15 971 (25.6)	4542 (20.8)	3602 (23.1)	4531 (29.5)	3296 (34.5)	17.9
Alcohol drinker (≥1 time/wk)	12 890 (20.7)	3382 (15.5)	3007 (19.3)	3559 (23.2)	2942 (30.8)	20.0
Baseline comorbidities						
Hypertension	37 287 (59.9)	13 224 (60.6)	9425 (60.6)	9034 (58.9)	5604 (58.7)	2.5
Diabetes	13 177 (21.2)	4400 (20.2)	3234 (20.8)	3353 (21.8)	2290 (22.9)	3.8
Dyslipidemia	31 559 (50.7)	10 593 (48.5)	8071 (51.9)	7901 (51.5)	4994 (52.3)	3.9
Chronic kidney disease	1625 (2.6)	627 (2.9)	397 (2.6)	376 (2.5)	225 (2.4)	1.7
Heart failure	7621 (12.2)	3099 (14.2)	1896 (12.2)	1695 (11.0)	931 (9.7)	7.5
Vascular disease	7.930 (12.7)	2808 (12.9)	1982 (12.7)	1929 (12.6)	1211 (12.7)	0.5
Prior ischemic stroke or TIA	10 227 (16.4)	3900 (17.9)	2532 (16.3)	2437 (15.9)	1358 (14.2)	5.2
COPD	7479 (12.0)	2930 (13.4)	1852 (11.9)	1741 (11.34)	956 (10.0)	5.6
Malignant neoplasm	9968 (16.0)	3345 (15.3)	2531 (16.32)	2443 (15.9)	1649 (17.3)	2.8
Laboratory findings, mean (SD), mg/dL						
Fasting glucose	106.1 (31.3)	105.9 (32.1)	105.8 (31.0)	106.2 (30.3)	107.2 (31.3)	2.4
Total cholesterol	196.8 (40.4)	197.7 (41.4)	197.7 (40.3)	195.8 (39.6)	194.7 (39.4)	4.6
LDL-C	116.4 (38.0)	117.4 (40.0)	116.9 (37.4)	115.4 (36.5)	114.4 (36.0)	4.7
HDL-C	53.4 (25.6)	53.8 (31.2)	53.1 (21.1)	53.2 (22.2)	53.5 (22.8)	1.6

Abbreviations: ASD, absolute standardized difference; BMI, body mass index; COPD, chronic obstructive pulmonary disease; HDL-C, high-density lipoprotein cholesterol; LDL-C, low-density lipoprotein cholesterol; MET, metabolic equivalent; TIA, transient ischemic attack.

SI conversion factors: To convert glucose to millimoles per liter, multiply by 0.0555; total cholesterol, HDL-C, and LDL-C to millimoles per liter, multiply by 0.0259.

^a^ High tertile of annual income was evaluated based on the total national health insurance premiums paid by the insured individual in the index year, which is proportional to the individual’s income.

### Physical Activity Level and All-Cause Dementia

**Figure 1.**
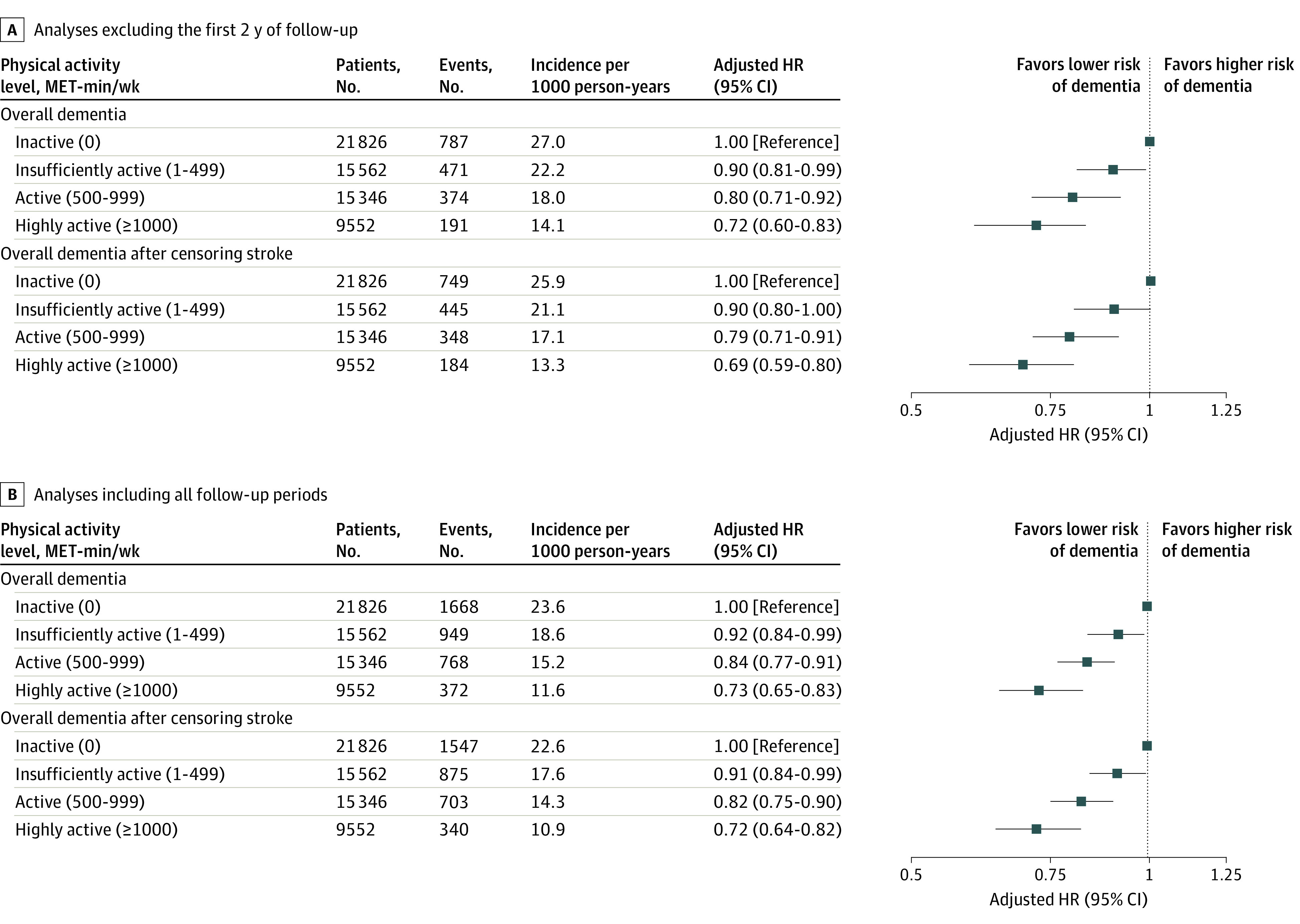
Risk of Overall Dementia in Association With Total Leisure-Time Physical Activity Level The first 2 years of follow-up were excluded to minimize reverse causation bias. Incident dementia occurring 2 years after enrollment was assessed. The model was adjusted for age, sex, body mass index, Hospital Frailty Risk score, annual income, smoking, alcohol, hypertension, diabetes mellitus, dyslipidemia, chronic kidney disease, heart failure, vascular disease, prior ischemic stroke or transient ischemic attack, chronic obstructive pulmonary disease, and malignancy. *P* < .001 for trend in all analyses. HR indicates hazard ratio; MET, metabolic equivalent.

During a median follow-up of 42 months (IQR, 27-55 months), 3757 participants (6.0%) had all-cause dementia. After excluding the first 2 years, 1823 participants developed dementia, with an overall incidence of 21.6 per 1000 person-years during follow-up. When stratified by physical activity level, the incidence was 27.0 per 1000 person-years in the inactive group, 22.2 per 1000 person-years in the insufficiently active group, 18.0 per 1000 person-years in the active group, and 14.1 per 1000 person-years in the highly active group (Figure 1A). The cumulative incidence of dementia was associated with a progressively decreasing trend with increasing physical activity level (*P* < .001 for trend) (eFigure 2 in the Supplement). In Fine-Gray competing-risk multivariable regression models, the insufficiently active (adjusted HR [HR], 0.90; 95% CI, 0.81-0.99), active (adjusted HR, 0.80; 95% CI, 0.71-0.92), and highly active (adjusted HR, 0.72; 95% CI, 0.60-0.83) groups were associated with a lower risk of dementia than the inactive group.

After censoring for stroke, activity levels of less than 1 to 499 MET-min/wk (insufficiently active group; HR, 0.90; 95% CI, 0.80-1.00), 500 to 999 MET-min/wk (active group; HR, 0.79; 95% CI, 0.71-0.91), and more than 1000 MET-min/wk (highly active group; HR, 0.69; 95% CI, 0.59-0.80) were associated with a lower risk of dementia than an activity level that included only basic movements (the inactive group), which was consistent with results of the main analysis. Moreover, analyses by including all follow-up periods coincided with the main findings (Figure 1B). In subgroup analysis, associations between physical activity level and risk of dementia were consistent regardless of age, sex, and comorbidities (eTable 3 in the Supplement).

**Figure 2.**
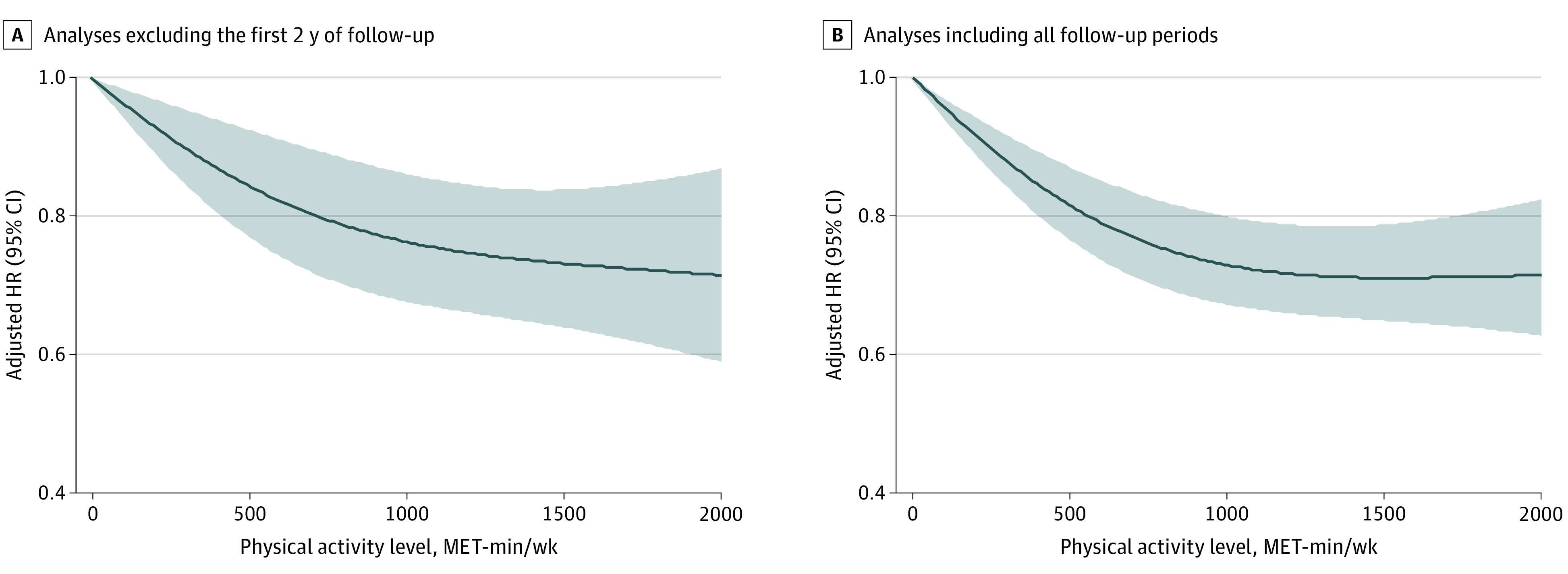
Association Between Risk of Dementia and Continuous Measures of Physical Activity Level The first 2 years of follow-up were excluded to minimize reverse causation bias. Incident dementia occurring 2 years after enrollment was assessed. A restricted cubic spline curve was constructed with regard to physical activity treated as a continuous variable. Hazard ratios were calculated with 0 MET-min/wk as a reference. A nonlinear association between physical activity and dementia risk was noted. The model was adjusted for age, sex, body mass index, Hospital Frailty Risk score, annual income, smoking, alcohol, hypertension, diabetes, dyslipidemia, chronic kidney disease, heart failure, vascular disease, prior ischemic stroke or transient ischemic attack, chronic obstructive pulmonary disease, and malignancy. HR indicates hazard ratio; MET, metabolic equivalent.

Figure 2 shows the association between risk of dementia and continuous measures of physical activity level using the restricted cubic spline curve. A nonlinear association between physical activity and risk of dementia was noted. The slope was steep (between 0 and 1000 MET-min/wk), reaching a plateau above 1500 MET-min/wk, wherein the association between the dementia and continuous measures of physical activity started with a low amount of total physical activity.

### Light-Intensity Physical Activity and Dementia

**Figure 3.**
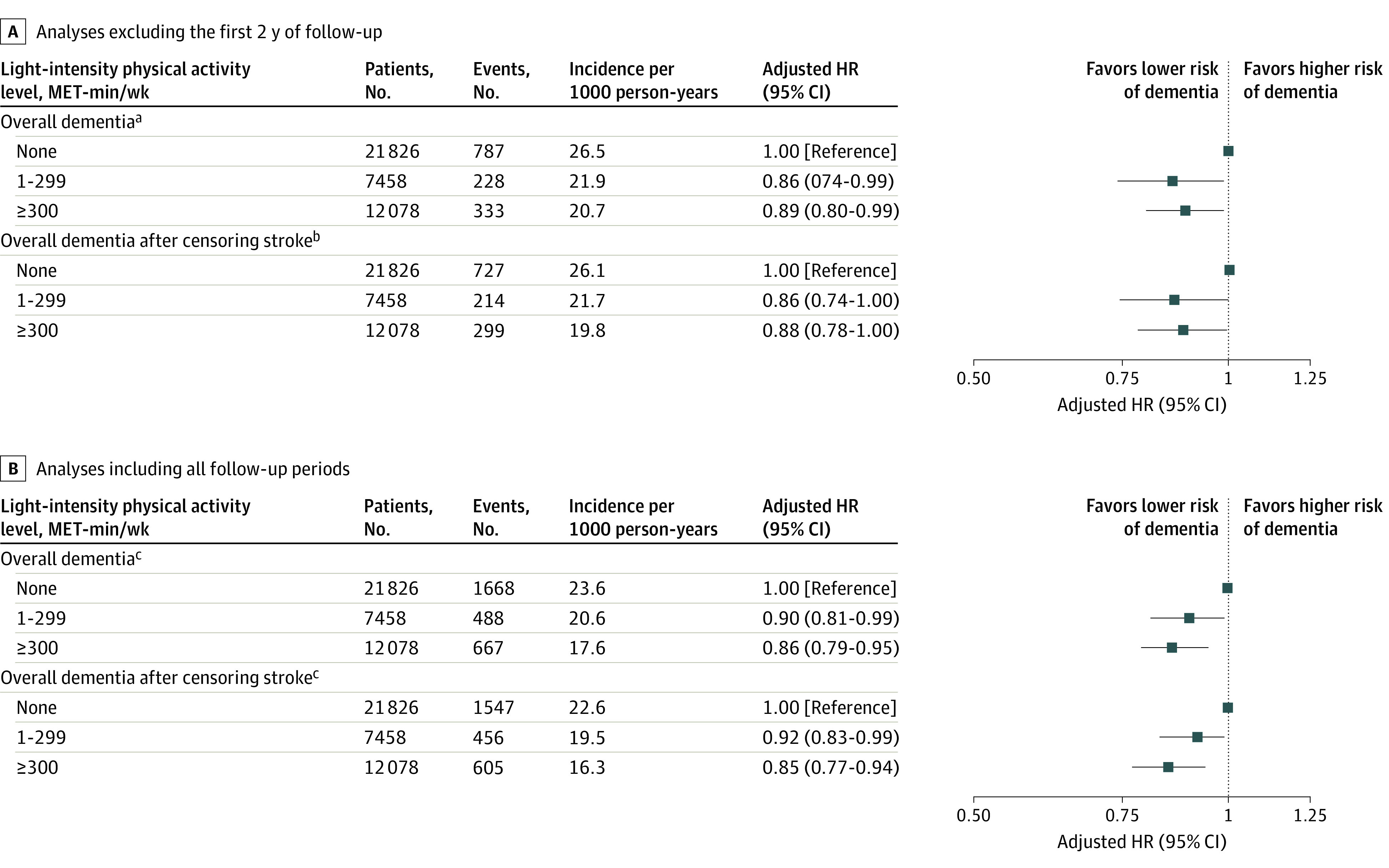
Risk of Overall Dementia in Association With the Light-Intensity Physical Activity Level The first 2 years of follow-up were excluded to minimize reverse causation bias. Incident dementia occurring 2 years after enrollment was assessed. Light-intensity physical activity was assessed in participants who did not perform activity beyond moderate-intensity physical activity (n = 41 362). The model was adjusted for age, sex, body mass index, Hospital Frailty Risk score, annual income, smoking, alcohol, hypertension, diabetes mellitus, dyslipidemia, chronic kidney disease, heart failure, vascular disease, prior ischemic stroke or transient ischemic attack, chronic obstructive pulmonary disease, and malignancy. HR indicates hazard ratio; MET, metabolic equivalent. ^a^*P* = .08 for trend. ^b^*P* = .05 for trend. ^c^*P* = .001 for trend.

**Figure 4.**
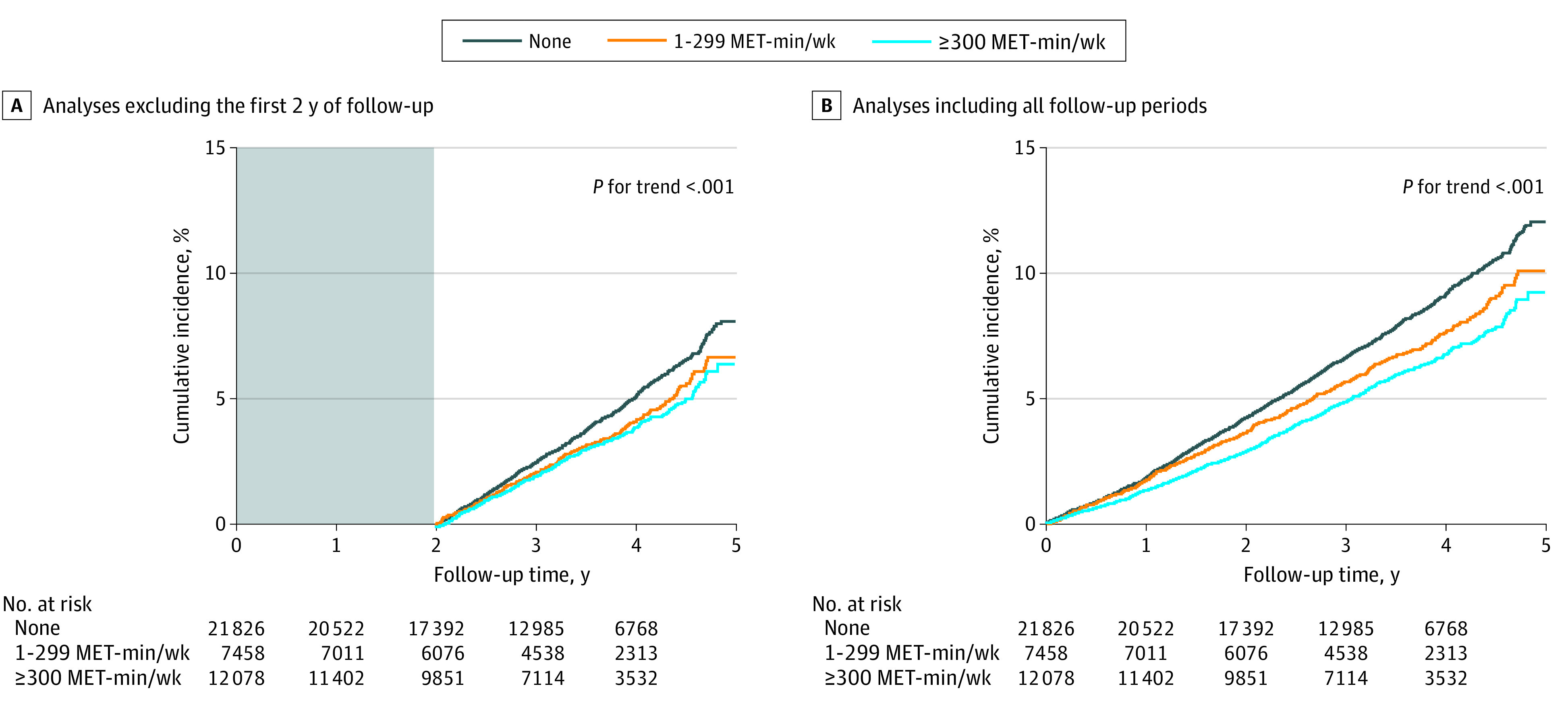
Cumulative Incidence Curve of Overall Dementia According to Light-Intensity Physical Activity The first 2 years of follow-up were excluded to minimize reverse causation bias. Incident dementia occurring 2 years after enrollment was assessed. Light-intensity physical activity was assessed in participants who did not perform activity beyond moderate-intensity physical activity (n = 41 362). MET indicates metabolic equivalent.

Among the 62 286 participants, 41 362 did not perform any activity beyond MPA, whereas 21 826 (52.8%) performed no LPA, 7458 (18.0%) performed LPA at a level of 1 to 299 MET-min/wk, and 12 078 (29.2%) participants performed LPA at a level of 300 or more MET-min/wk (Figure 3; eTable 4 in the Supplement). Cumulative incidence of dementia was associated with a progressively decreasing trend with increasing LPA level (*P* < .001 for trend; Figure 4). Furthermore, in comparison with total sedentary behavior, even a low amount of LPA (1-299 MET-min/wk; mean, 189 MET-min/wk) was associated with reduced risk of dementia (adjusted HR, 0.86; 95% CI, 0.74-0.99). On inclusion of all follow-up periods, LPA levels of 1 to 299 MET-min/wk (adjusted HR, 0.90; 95% CI, 0.81-0.99) and 300 or more MET-min/wk (adjusted HR, 0.86; 95% CI, 0.79-0.95) were associated with a progressively lower risk of dementia than no LPA (total sedentary behavior) (Figure 3B). This association was consistently observed even after censoring for stroke.

### Physical Activity Level and Dementia Subtype

The incidence of AD was 19.9 per 1000 person-years in the inactive group, 17.1 per 1000 person-years in the insufficiently active group, 13.1 per 1000 person-years in the active group, and 10.9 per 1000 person-years in the highly active group (eFigures 3 and 4 in the Supplement). Cumulative incidence of AD was associated with a progressively decreasing trend with increasing physical activity level (*P* < .001 for trend) (eFigure 5 in the Supplement). In the Fine-Gray competing risk regression models, activity levels of 500 to 999 MET-min/wk (active group; adjusted HR, 0.82; 95% CI, 0.71-0.95) and more than 1000 MET-min/wk (highly active group; adjusted HR, 0.78; 95% CI, 0.64-0.93) were associated with a lower risk of AD than activity that included only basic movements (inactive group). Incidence of vascular dementia was 3.3 per 1000 person-years in the inactive group, 2.5 per 1000 person-years in the insufficiently active group, 2.4 per 1000 person-years in the activity group, and 1.7 per 1000 person-years in the highly active group. An inverse dose-response association was not observed between vascular dementia and physical activity level (eFigures 3, 4, and 6 in the Supplement). After including all follow-up periods or censoring for stroke, the association between physical activity and risk of dementia subtype was consistent with the primary analyses.

### Sensitivity Analysis

In sensitivity analyses excluding patients with a total KDSQ score of 4 or higher (n = 2220), a progressively decreasing dementia risk trend with increasing physical activity level (*P* < .001 for trend) was consistent with the main analyses (eFigure 7 in the Supplement). Sensitivity analyses by time-varying regression were also consistent with the main analyses (eFigure 8 in the Supplement).

## Discussion

In this nationwide study using a large real-world Asian cohort, our principal findings were as follows: (1) increased physical activity level was associated with a reduced risk of dementia in older adults, (2) the association between physical activity and reduced risk of dementia started with a low amount of total physical activity, and (3) even a low amount of LPA correlated with a reduced risk of dementia in older adults.

### Dose-Response Association Between Physical Activity and Dementia

Because approximately 40 million people are affected with dementia worldwide and this number is expected to increase with an increasing aged population,^4^ we assessed the dose-response association between physical activity and dementia. We calculated the total amount of physical activity as a continuous variable. Consequently, this association started with a low total physical activity amount (Figure 2). This finding suggests that even a low amount of physical activity, as opposed to total sedentary behavior, could lead to a reduction in risk of dementia.

### LPA and Dementia

Although previous guidelines recommended only MPA or VPA in older adults, recent studies^7^ have shown that LPA was associated with an improvement in cardiometabolic health and mortality risk reduction. Another study^36^ also demonstrated that total sedentary behavior was associated with unfavorable health outcomes. Hence, the recently published World Health Organization 2020 guidelines^3^ on physical activity recommend replacing sedentary time with physical activity of any intensity, including LPA. However, some studies^8,20^ regarding LPA and cognitive function had a small number of participants and showed inconsistent results.

Our study showed that 66.4% (41 362 of 62 286) of older adult participants did not perform any activity beyond MPA, and an increased LPA level in these participants was associated with a lower risk of dementia. Physical exercises for older adults should be designed according to their age, functional capacity, aging trajectories, comorbidities, safety, and previous experience of exercise. Therefore, older adults who cannot perform activity beyond MPA because of their frailty and comorbidities could benefit from LPA. Replacing sedentary time with LPA may help decrease dementia risk in older adults.

### Types of Dementia

Previous studies^37,38^ have shown that physical activity was more protective against AD than against vascular dementia. Our results were consistent with findings from previous studies. An inverse dose-response association was not observed between vascular dementia and physical activity level in our studies. These findings suggest that other modifiable risk factors, such as hypertension and diabetes mellitus, could play an important role in vascular dementia. Thus, further studies exploring these risk factors are needed.

### Benefits and Importance of Physical Activity

The World Health Organization 2020 guidelines on physical activity emphasized the various benefits of physical activity.^3^ Physical activity was not only associated with improved cardiovascular health but also with improvement in mental health and a lower incidence of cancer.^39,40^ Moreover, Jin et al^28^ recently reported that the key target range of physical activity was associated with maximum benefits in reducing atrial fibrillation risk. The current European atrial fibrillation guidelines recommend that adequate physical activity should be considered to reduce incidence or prevent recurrence of atrial fibrillation.^41^ Our study showed an association between increased physical activity and a reduced risk of dementia.

### Controversy Regarding Effect of Physical Activity on Dementia

The effect of physical activity on dementia remains controversial. Some studies have suggested that a lower risk of dementia in physically active people could be attributed to reverse causation or short follow-up duration.^15,16,17,18,19^ Because our study is an observational cohort study, it cannot be used to establish causal relationships, and residual confounding is likely to persist. In addition, the possibility of reverse causation still exists despite sensitivity analyses.

### Strengths and Limitations

Despite the controversy concerning physical activity and dementia, this study had certain strengths, including the large number of older adults with available data regarding physical activity. We also assessed the dose-response association between physical activity and dementia, focusing on the association of LPA with the incidence of dementia. Various sensitivity analyses (ie, censoring for stroke, excluding the first 2 years of follow-up, excluding patients with a total KDSQ score ≥4, or time-varying regression) revealed consistent findings, further supporting our main result.

This study also had several imitations. First, studies using administrative databases are susceptible to errors arising from coding inaccuracies. To minimize this problem, we applied definitions that had already been validated in previous studies using the Korean NHIS cohort.^29,42^ Second, we used self-reported data for physical activity, collected at a single time point. The conditions at the time of questionnaire completion may not represent the actual lifetime physical activity conditions. Behavioral changes during the follow-up period were also unmeasurable. Furthermore, in the self-report–structured questionnaires used for Korean health checkups, there was no mention of resistance exercise or muscle-strengthening activities. Third, the dementia outcome was ascertained by clinical diagnosis and associated medication use (a high-specificity assessment method). Therefore, milder cases might have gone undetected because of low sensitivity. Fourth, our study population was older than that of other studies, and the follow-up duration was short. Because dementia has a long preclinical period, our findings might have been affected by reverse causation bias despite various sensitivity analyses. Fifth, our study population comprised only Asian individuals, whose overall body mass index is lower than that of the US population, thus limiting generalizability.^43^ Sixth, using VPA as a proxy for determining health status in adults can be a limitation. There may be some adults who chose to do MPA over VPA who are just as healthy. The evidence of the benefit of physical activity to health is based on moderate-intensity equivalence, meaning the benefit from someone who does 15 minutes of VPA is similar to that of someone who performs 30 minutes of MPA.

## Conclusions

The findings of this cohort study indicated that increased physical activity among older adults, including a low amount of LPA, was associated with a reduced risk of dementia. Promotion of LPA might reduce the risk of dementia in older adults.

## Notes

Supplement. **eMethods.** Supplemental Methods
eReferences
**eTable 1.** Definitions of Comorbidities **eTable 2.** List of 109 Variables Contributing to Calculating the Hospital Frailty Risk Score **eTable 3.** Association Between Physical Activity Level and Risk of Dementia According to Subgroup **eTable 4.** Baseline Characteristics Stratified by the Light-Intensity Physical Activity Level **eFigure 1.** Standardized Self-Report Questionnaires Regarding Physical Activity in Korean Health Check-up Data **eFigure 2.** Cumulative Incidence Curve of Overall Dementia According to Leisure-Time Physical Activity **eFigure 3.** Risk of Dementia Sub-type in Relation to the Total Leisure-Time Physical Activity Level by Excluding the First 2 Years of Follow-up **eFigure 4.** Risk of Dementia Sub-type in Relation to the Total Leisure-Time Physical Activity Level by Including All Follow-up Period **eFigure 5.** Cumulative Incidence Curve of Alzheimer’s Dementia According to Leisure-Time Physical Activity **eFigure 6.** Cumulative Incidence Curve of Vascular Dementia According to Leisure-Time Physical Activity **eFigure 7.** Risk of Overall Dementia in Relation to the Total Leisure-Time Physical Activity Level by Excluding KDSQ Score ≥4 (Total N = 60,066) **eFigure 8.** Risk of Overall Dementia in Relation to the Total Leisure-Time Physical Activity Level by Time-Varying Regression Analyses
