# Supplementary material for: Association of Physical Activity Level With Risk of Dementia in a Nationwide Cohort in Korea
Source: JAMA Netw Open. 2021 Dec 16;4(12):e2138526. doi: 10.1001/jamanetworkopen.2021.38526 (PMC8678703; doi:10.1001/jamanetworkopen.2021.38526)
Supplement: Supplement. — eMethods. Supplemental Methods eReferences eTable 1. Definitions of Comorbidities eTable 2. List of 109 Variables Contributing to Calculating the Hospital Frailty Risk Score eTable 3. Association Between Physical Activity Level and Risk of Dementia According to Subgroup eTable 4. Baseline Characteristics Stratified by the Light-Intensity Physical Activity Level eFigure 1. Standardized Self-Report Questionnaires Regarding Physical Activity in Korean Health Check-up Data eFigure 2. Cumulative Incidence Curve of Overall Dementia According to Leisure-Time Physical Activity eFigure 3. Risk of Dementia Sub-type in Relation to the Total Leisure-Time Physical Activity Level by Excluding the First 2 Years of Follow-up eFigure 4. Risk of Dementia Sub-type in Relation to the Total Leisure-Time Physical Activity Level by Including All Follow-up Period eFigure 5. Cumulative Incidence Curve of Alzheimer’s Dementia According to Leisure-Time Physical Activity eFigure 6. Cumulative Incidence Curve of Vascular Dementia According to Leisure-Time Physical Activity eFigure 7. Risk of Overall Dementia in Relation to the Total Leisure-Time Physical Activity Level by Excluding KDSQ Score ≥4 (Total N = 60,066) eFigure 8. Risk of Overall Dementia in Relation to the Total Leisure-Time Physical Activity Level by Time-Varying Regression Analyses [file jamanetwopen-e2138526-s001.pdf]

Supplementary Online Content

Yoon M, Yang PS, Jin MN, et al. Association of physical activity level with risk of dementia in a nationwide cohort in Korea. *JAMA Netw Open*. 2021;4(12):e2138526. doi:10.1001/jamanetworkopen.2021.38526

eMethods. Supplemental Methods

eReferences

eTable 1. Definitions of Comorbidities

eTable 2. List of 109 Variables Contributing to Calculating the Hospital Frailty Risk Score

eTable 3. Association Between Physical Activity Level and Risk of Dementia According to Subgroup

eTable 4. Baseline Characteristics Stratified by the Light-Intensity Physical Activity Level

eFigure 1. Standardized Self-Report Questionnaires Regarding Physical Activity in Korean Health Check-up Data

eFigure 2. Cumulative Incidence Curve of Overall Dementia According to Leisure-Time Physical Activity

eFigure 3. Risk of Dementia Sub-type in Relation to the Total Leisure-Time Physical Activity Level by Excluding the First 2 Years of Follow-up

eFigure 4. Risk of Dementia Sub-type in Relation to the Total Leisure-Time Physical Activity Level by Including All Follow-up Period

eFigure 5. Cumulative Incidence Curve of Alzheimer's Dementia According to Leisure-Time Physical Activity

eFigure 6. Cumulative Incidence Curve of Vascular Dementia According to Leisure-Time Physical Activity

eFigure 7. Risk of Overall Dementia in Relation to the Total Leisure-Time Physical Activity Level by Excluding KDSQ score ≥4 (Total N = 60,066)

eFigure 8. Risk of Overall Dementia in Relation to the Total Leisure-Time Physical Activity Level by Time-Varying Regression Analyses

This supplementary material has been provided by the authors to give readers additional information about their work.

© 2021 Yoon M et al. *JAMA Network Open*.

eMethods. Supplemental Methods

KDSQ consists of questions for global memory function and instrumental activities of daily living, including five items that could detect early changes in cognitive decline.[1] Each item on the KDSQ is scored from 0 to 2, with a higher score indicating poor cognitive function. Total KDSQ score ≥4 indicates possible cognitive impairment, which need further investigation to confirm cognitive dysfunction. The KDSQ is not influenced by educational level or age and has shown a 0.79 sensitivity and 0.80 specificity for predicting dementia.[1, 2]

eReferences

1. Lee SJ, Han JH, Hwang JW, Paik JW, Han C, Park MH. Screening for Normal Cognition, Mild Cognitive Impairment, and Dementia with the Korean Dementia Screening Questionnaire. *Psychiatry Investig.* 2018;15(4):384-389. doi:10.30773/pi.2017.08.24

2. Jeon Y, Yun K, Kim Y. Validation of KDSQ-P as selecting elderly for KDSQ-C. *Korean J Health Promot.* 2010;10(2):45-52

© 2021 Yoon M et al. *JAMA Network Open*.

eTable 1. Definitions of comorbidities

Comorbidities	Definitions	ICD-10 codes or conditions
Hypertension	Defined from diagnosis*	I10, I11, I12, I13, I15 and antihypertensive medication
Diabetes mellitus	Defined from diagnosis*<br>plus treatment	E10, E11, E12, E13, E14<br>Treatment: all kinds of oral anti-diabetics and insulin
Dyslipidemia	Defined from diagnosis*	ICD-10: E78
Chronic kidney disease	Defined from eGFR or diagnosis*<br>(if laboratory value was not available, diagnosis code was used)	eGFR <60mL/min per 1.73 m^2<br>ICD-10: N18, N19
Heart failure	Defined from diagnosis*	I11.0, I50, I97.1
Vascular disease	Defined from diagnosis*	I21, I22, I25.2<br>I70.0, I70.1, I70.2, I70.8, I70.9
Previous ischemic stroke	Defined from diagnosis*	I63, I64
Transient ischemic attack (TIA)	Defined from diagnosis*	G45
Chronic obstructive pulmonary disease (COPD)	Defined from diagnosis* plus treatment	J42, J43(except J43.0), J44<br>Treatment: SABA, SAMA, LABA, LAMA, ICS, ICS+LABA, or methylxanthine (>1 months).
Malignancy	Defined from diagnoses *	C00-C97

*To ensure accuracy, comorbidities were established based on one inpatient or two outpatient records of ICD-10 codes in the database.
eGFR: Estimated glomerular filtration rate; ICD-10: International Classification of Diseases-10th Revision.

© 2021 Yoon M et al. *JAMA Network Open*.

eTable 2. List of 109 variables contributing to calculating the Hospital Frailty Risk Score

ICD-10 Description	ICD-10 code	Points
Dementia in Alzheimer's disease	F00	7.1
Hemiplegia	G81	4.4
Alzheimer's disease	G30	4
Sequelae of cerebrovascular disease (secondary codes)	I69	3.7
Other symptoms and signs involving the nervous and musculoskeletal systems (R29.6 Tendency to fall)	R29	3.6
Other disorders of urinary system (includes urinary tract infection and urinary incontinence)	N39	3.2
Superficial injury of head	S00	3.2
Delirium, not induced by alcohol and other psychoactive substances	F05	3.2
Unspecified fall	W19	3.2
Unspecified hematuria	R31	3
Other bacterial agents as the cause of diseases classified to other chapters (secondary code)	B96	2.9
Other symptoms and signs involving cognitive functions and awareness	R41	2.7
Other cerebrovascular diseases	I67	2.6
Convulsions, not elsewhere classified	R56	2.6
Abnormalities of gait and mobility	R26	2.6
Somnolence, stupor and coma	R40	2.5
Intracranial injury	S06	2.4
Complications of genitourinary prosthetic devices, implants and grafts	T83	2.4
Other disorders of fluid, electrolyte and acid base balance	E87	2.3
Other joint disorders, not elsewhere classified	M25	2.3
Volume depletion	E86	2.3
Fracture of shoulder and upper arm	S42	2.3
Senility	R54	2.2
Unspecified dementia	F03	2.1
Care involving use of rehabilitation procedures	Z50	2.1
Other fall on same level	W18	2.1
Cellulitis	L03	2
Vascular dementia	F01	2
Superficial injury of lower leg	S80	2
Problems related to medical facilities and other health care	Z75	2
Deficiency of other B group vitamins	E53	1.9
Blindness and low vision	H54	1.9
Other functional intestinal disorders	K59	1.8
Fracture of rib(s), sternum and thoracic spine	S22	1.8
Syncope and collapse	R55	1.8
Acute renal failure	N17	1.8
Parkinson's disease	G20	1.8
Problems related to social environment	Z60	1.8
Decubitus ulcer	L89	1.7
Carrier of infectious disease	Z22	1.7
Streptococcus and staphylococcus as the cause of diseases classified to other chapters	B95	1.7
Other septicemia	A41	1.6
Duodenal ulcer	K26	1.6
Hypotension	I95	1.6
Unspecified renal failure	N19	1.6
Ulcer of lower limb, not elsewhere classified	L97	1.6
Other symptoms and signs involving general sensations and perceptions	R44	1.6

© 2021 Yoon M et al. *JAMA Network Open*.

**ICD-10 Description**	**ICD-10 code**	**Points**
Epilepsy	G40	1.5
Other arthrosis	M19	1.5
Respiratory failure, not elsewhere classified	J96	1.5
Personal history of other diseases and conditions	Z87	1.5
Exposure to unspecified factor	X59	1.5
Osteoporosis without pathological fracture	M81	1.4
Abnormal results of function studies	R94	1.4
Fracture of lumbar spine and pelvis	S32	1.4
Chronic renal failure	N18	1.4
Fracture of femur	S72	1.4
Other disorders of pancreatic internal secretion	E16	1.4
Other disorders of kidney and ureter, not elsewhere classified	N28	1.3
Retention of urine	R33	1.3
Unknown and unspecified causes of morbidity	R69	1.3
Transient cerebral ischemic attacks and related syndromes	G45	1.2
Other degenerative diseases of nervous system, not elsewhere classified	G31	1.2
Unspecified urinary incontinence	R32	1.2
Symptoms and signs involving emotional state	R45	1.2
Other and unspecified injuries of head	S09	1.2
Nosocomial condition	Y95	1.2
Pneumonia, organism unspecified	J18	1.1
Diarrhea and gastroenteritis of presumed infectious origin	A09	1.1
Other soft tissue disorders, not elsewhere classified	M79	1.1
Open wound of head	S01	1.1
Other bacterial intestinal infections	A04	1.1
Fall involving bed	W06	1.1
Problems related to care-provider dependency	Z74	1.1
Speech disturbances, not elsewhere classified	R47	1
Pneumonitis due to solids and liquids	J69	1
Artificial opening status	Z93	1
Vitamin D deficiency	E55	1
Gangrene, not elsewhere classified	R02	1
Thyrotoxicosis [hyperthyroidism]	E05	0.9
Symptoms and signs concerning food and fluid intake	R63	0.9
Other hearing loss	H91	0.9
Scoliosis	M41	0.9
Fall on same level from slipping, tripping and stumbling	W01	0.9
Fall on and from stairs and steps	W10	0.9
Cerebral Infarction	I63	0.8
Other diseases of digestive system	K92	0.8
Dysphagia	R13	0.8
Osteoporosis with pathological fracture	M80	0.8
Agent resistant to penicillin and related antibiotics	U80	0.8
Dependence on enabling machines and devices	Z99	0.8
Abnormalities of heart beat	R00	0.7
Calculus of kidney and ureter	N20	0.7
Mental and behavioral disorders due to use of alcohol	F10	0.7
Unspecified acute lower respiratory infection	J22	0.7
Other medical procedures as the cause of abnormal reaction of the patient	Y84	0.7
Other abnormal findings of blood chemistry	R79	0.6
Problems related to life-management difficulty	Z73	0.6
Spinal stenosis (secondary code only)	M48	0.5
Depressive episode	F32	0.5
Open wound of forearm	S51	0.5
Personal history of risk-factors, not elsewhere classified	Z91	0.5

© 2021 Yoon M et al. *JAMA Network Open*.

ICD-10 Description	ICD-10 code	Points
Other anemia	D64	0.4
Disorders of mineral metabolism	E83	0.4
Polyarthrosis	M15	0.4
Other local infections of skin and subcutaneous tissue	L08	0.4
Nausea and vomiting	R11	0.3
Other noninfective gastroenteritis and colitis	K52	0.3
Fever of unknown origin	R50	0.1

ICD-10, International Classification of Diseases-10th Revision.

© 2021 Yoon M et al. *JAMA Network Open*.

eTable 3. Association between physical activity level and risk of dementia according to subgroup

Physical activity level (MET-min/week)	Inactive (0)	Insufficiently active (1-499)	Active (500-999)	Highly active (≥1,000)	
Characteristics	Adjusted hazard ratio* (95% confidence interval)				*P* for interaction
Age					0.08
65-74 years	1.00 (ref.)	0.94 (0.83-1.06)	0.85 (0.75-0.97)	0.71 (0.60-0.84)	
75-84 years	1.00 (ref.)	0.84 (0.75-0.95)	0.80 (0.70-0.91)	0.74 (0.61-0.89)	
≥85 years	1.00 (ref.)	1.11 (0.89-1.40)	0.87 (0.64-1.18)	1.13 (0.75-1.69)	
Sex					0.21
Male	1.00 (ref.)	0.91 (0.78-1.04)	0.84 (0.72-0.98)	0.66 (0.55-0.78)	
Female	1.00 (ref.)	0.91 (0.83-1.00)	0.83 (0.76-0.93)	0.79 (0.68-0.92)	
BMI					0.58
≥25 kg/m^2	1.00 (ref.)	0.98 (0.85-1.13)	0.81 (0.69-0.95)	0.74 (0.60-0.91)	
<25 kg/m^2	1.00 (ref.)	0.92 (0.82-1.02)	0.83 (0.73-0.93)	0.68 (0.56-0.79)	
Hospital Frailty Risk score					0.70
Score ≥5	1.00 (ref.)	0.94 (0.86-1.03)	0.81 (0.74-0.89)	0.75 (0.67-0.85)	
Score <5	1.00 (ref.)	0.72 (0.59-0.88)	0.85 (0.69-1.01)	0.50 (0.35-0.70)	
Income					0.87
High	1.00 (ref.)	0.92 (0.82-1.03)	0.84 (0.75-0.97)	0.79 (0.67-0.93)	
Low-intermediate	1.00 (ref.)	0.88 (0.80-0.97)	0.86 (0.78-0.96)	0.71 (0.61-0.81)	
Smoking					0.06
Yes	1.00 (ref.)	0.83 (0.69-1.00)	0.81 (0.68-0.98)	0.61 (0.48-0.77)	
No	1.00 (ref.)	0.93 (0.85-1.00)	0.85 (0.77-0.94)	0.75 (0.66-0.86)	
Alcohol					0.11
Yes	1.00 (ref.)	0.72 (0.58-0.90)	0.84 (0.68-1.04)	0.57 (0.44-0.74)	
No	1.00 (ref.)	0.94 (0.86-1.03)	0.83 (0.76-0.92)	0.75 (0.66-0.85)	

© 2021 Yoon M et al. *JAMA Network Open*.

Physical activity level (MET-min/week)	Inactive (0)	Insufficiently active (1-499)	Active (500-999)	Highly active (≥1,000)	
Hypertension					0.33
Yes	1.00 (ref.)	0.88 (0.80-0.97)	0.85 (0.76-0.94)	0.73 (0.64-0.84)	
No	1.00 (ref.)	0.97 (0.84-1.11)	0.85 (0.73-0.99)	0.68 (0.55-0.83)	
Diabetes mellitus					0.43
Yes	1.00 (ref.)	0.90 (0.77-1.06)	0.50 (0.72-1.00)	0.76 (0.62-0.95)	
No	1.00 (ref.)	0.91 (0.83-1.00)	0.85 (0.77-0.94)	0.69 (0.60-0.79)	
Dyslipidemia					0.49
Yes	1.00 (ref.)	0.92 (0.82-1.03)	0.85(0.75-0.96)	0.67 (0.57-0.78)	
No	1.00 (ref.)	0.90 (0.80-1.00)	0.84 (0.74-0.96)	0.77 (0.65-0.90)	
Chronic kidney disease					0.62
Yes	1.00 (ref.)	0.78 (0.51-1.21)	1.04 (0.68-1.58)	0.68 (0.53-1.12)	
No	1.00 (ref.)	0.91 (0.84-0.99)	0.84 (0.77-0.92)	0.71 (0.64-0.80)	
Heart failure					0.10
Yes	1.00 (ref.)	0.96 (0.80-1.16)	0.83 (0.68-1.02)	0.86 (0.65-1.13)	
No	1.00 (ref.)	0.90 (0.82-0.98)	0.85 (0.77-0.94)	0.69 (0.61-0.78)	
Vascular disease					0.99
Yes	1.00 (ref.)	0.87 (0.71-1.07)	0.91 (0.73-1.12)	0.58 (0.43-0.80)	
No	1.00 (ref.)	0.92 (0.84-0.99)	0.83 (0.76-0.92)	0.74 (0.65-0.83)	
Prior ischemic stroke or TIA					0.71
Yes	1.00 (ref.)	0.87 (0.74-1.01)	0.86 (0.73-01.02)	0.60 (0.47-0.77)	
No	1.00 (ref.)	0.93 (0.84-1.02)	0.84 (0.76-0.94)	0.75 (0.66-0.85)	
COPD					0.62
Yes	1.00 (ref.)	0.98 (0.81-1.19)	0.91 (0.73-1.12)	0.65 (0.47-0.89)	
No	1.00 (ref.)	0.90 (0.82-0.98)	0.83 (0.76-0.92)	0.72 (0.64-0.81)	
Malignancy					0.25

© 2021 Yoon M et al. *JAMA Network Open*.

Physical activity level (MET-min/week)	Inactive (0)	Insufficiently active (1-499)	Active (500-999)	Highly active (≥1,000)	
Yes	1.00 (ref.)	0.97 (0.79-1.18)	1.00 (0.82-1.23)	0.74 (0.56-0.96)	
No	1.00 (ref.)	0.90 (0.82-0.98)	0.82 (0.74-0.90)	0.71 (0.62-0.80)	

*The model was adjusted for age, sex, BMI, income, smoking, alcohol, hypertension, diabetes mellitus, dyslipidemia, chronic kidney disease, heart failure, vascular disease, prior ischemic stroke or TIA, COPD, and malignancy.
BMI, body mass index; COPD, chronic obstructive pulmonary disease; MET, metabolic equivalent task; TIA, transient ischemic attack.

© 2021 Yoon M et al. *JAMA Network Open*.

eTable 4. Baseline characteristics stratified by the light-intensity physical activity level

		Light-intensity physical activity level (MET-min/week)			
**Characteristic**	**All (N = 41,362)**	**None (N = 21,826)**	**1-299 (N = 7,458)**	**≥300 (N = 12,078)**	**ASD (%)**
Age (years)	73.6 ± 5.6	74.1 ± 5.9	73.6 ± 5.5	73.0 ± 5.0	13.6
Age groups:					13.0
65-74 years	26,738 (64.6)	13,351 (62.0)	4,810 (64.5)	8,397 (69.5)	
75-84 years	14,624 (35.4)	6,938 (31.8)	2,302 (30.9)	3,359 (27.8)	
≥85 years	2,025 (4.9)	1,357 (6.2)	346 (4.6)	322 (2.7)	
Males	14,370 (34.7)	7,317 (33.5)	2,295 (30.8)	4,758 (39.4)	12.1
BMI (kg/m^2)	23.8 ± 3.5	23.8 ± 3.6	23.9 ± 3.4	23.8 ± 3.3	3.4
Waist circumference (cm)	83.1 ± 9.0	83.0 ± 9.2	83.3 ± 8.8	83.1 ± 8.7	2.8
Systolic BP (mmHg)	13189 ± 17.2	131.9 ± 17.4	131.4 ± 16.8	131.8 ± 17.0	2.2
Diastolic BP (mmHg)	78.4 ± 10.6	78.6 ± 10.7	78.3 ± 10.4	78.3 ± 10.4	1.8
Hospital Frailty Risk score	0 (0–1.9)	0 (0–2.3)	0 (0–1.8)	0 (0–1.7)	9.6
High tertile of income	18,953 (45.8)	9,677 (44.3)	3,606 (48.4)	5,670 (46.9)	5.4
Smoker (Ex or current)	9,424 (22.8)	4,542 (20.8)	1,584 (21.2)	3,298 (27.3)	10.2
Alcohol drinker (≥1 time/week)	6,992 (16.9)	3,382 (15.5)	1,203 (16.1)	2,407 (19.9)	7.8
Baseline co-morbidities					
Hypertension	24,987 (60.4)	13,224 (60.6)	4,560 (61.1)	7,203 (59.6)	2.1
Diabetes mellitus	8,647 (20.9)	4,400 (20.2)	1,548 (20.8)	2,699 (22.3)	3.6
Dyslipidemia	20,655 (49.9)	10,593 (48.5)	3,868 (51.9)	6,194 (51.3)	4.4
Chronic kidney disease	1,152 (2.8)	627 (2.9)	203 (2.7)	322 (2.7)	0.8
Heart failure	5,498 (13.3)	3,099 (14.2)	1,002 (13.4)	1,397 (11.6)	5.2
Vascular disease	5,306 (12.8)	2,808 (12.9)	936 (12.6)	1,562 (12.9)	0.8
Prior ischemic stroke or TIA	7,149 (17.3)	3,900 (17.9)	1,236 (16.6)	2,013 (16.7)	2.3
COPD	5,240 (13.7)	2,930 (13.4)	891 (11.9)	1,419 (11.7)	3.4
Malignancy	6,469 (15.6)	3,345 (15.3)	1,198 (16.1)	1,926 (15.9)	1.4

© 2021 Yoon M et al. *JAMA Network Open*.

Laboratory findings					
Fasting glucose (mg/dL)	105.9 ± 31.5	105.9 ± 32.1	105.7 ± 31.5	106.2 ± 30.5	1.1
Total cholesterol (mg/dL)	197.3 ± 40.9	197.7 ± 41.4	198.0 ± 40.9	196.1 ± 40.0	3.2
LDL cholesterol (mg/dL)	117.0 ± 38.9	117.5 ± 40.0	117.1 ± 37.9	116.2 ± 37.3	2.3
HDL cholesterol (mg/dL)	53.4 ± 27.5	53.8 ± 31.2	52.9 ± 23.2	53.0 ± 22.2	2.3

All values are presented as number (%), mean ± standard deviation, or median (interquartile range)
Low-intensity physical activity was assessed in participants who did not perform activity beyond moderate intensity physical activity (n = 41,362).
ASD, absolute standardized difference; BMI, body mass index; BP, blood pressure; COPD, chronic obstructive pulmonary disease; HDL, high-density lipoprotein; LDL, low-density lipoprotein; MET, metabolic equivalent task; TIA, transient ischemic attack.

© 2021 Yoon M et al. *JAMA Network Open*.

eFigure 1. Standardized self-report questionnaires regarding physical activity in Korean health check-up data

[Annex No. 1] <Back>

※ These are questions about exercising.

6. These are questions about your physical activity for the last week. Please answer the following questions by ticking the appropriate box.

6-1. During the last week, how many days did you exercise vigorously for over 20 minutes until you were almost out of breath? (example: running, aerobics, high-speed cycling, mountain hiking, etc.)

☐ 0 ☐ 1 ☐ 2 ☐ 3 ☐ 4 ☐ 5 ☐ 6 ☐ 7

6-2. During the last week, how many days did you exercise in a moderate level for more than 30 minutes until you had to breathe a little faster than usual? (example: fast walking, tennis, bicycle riding, cleaning, etc.) ※ Except the relevant answer from 6-1

☐ 0 ☐ 1 ☐ 2 ☐3 ☐ 4 ☐ 5 ☐ 6 ☐ 7

6-3. During the last week, how many days did you walk for a total of 30 minutes or more in a day, including separate 10-minute walks? (example: light exercise, walk for work or for leisure, etc.)

※ Please exclude exercises you answered in 6-1 and 6-2

☐ 0 ☐ 1 ☐ 2 ☐ 3 ☐ 4 ☐ 5 ☐ 6 ☐ 7

© 2021 Yoon M et al. *JAMA Network Open*.

eFigure 2. Cumulative incidence curve of overall dementia according to leisure-time physical activity

(A) Excluding the first 2 years of follow-up

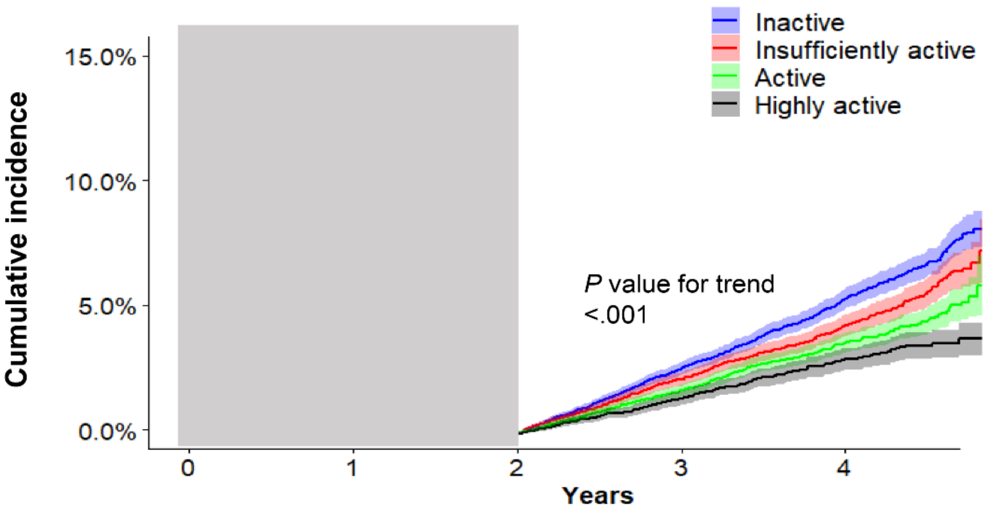

Number at risk

Inactive	21826	20824	17393	13393	7193
Insufficiently active	15562	15085	12738	9933	5257
Active	15346	14959	12687	9838	5042
Highly active	9552	9357	8062	6455	3443

(B) Including all follow-up period

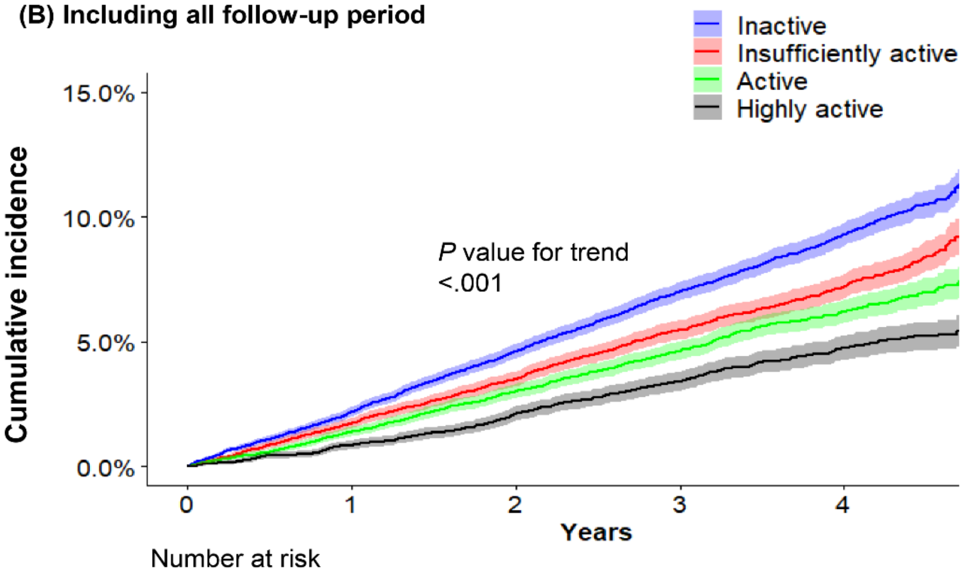

Number at risk

Inactive	21826	20824	17393	13393	7193
Insufficiently active	15562	15085	12738	9933	5257
Active	15346	14959	12687	9838	5042
Highly active	9552	9357	8062	6455	3443

(A) Analyses by excluding the first 2 years of follow-up to minimize reverse causation bias. Incident dementia occurring 2 years after enrollment was assessed. (B) Analyses by including all follow-up period.

© 2021 Yoon M et al. *JAMA Network Open*.

eFigure 3. Risk of dementia sub-type in relation to the total leisure-time physical activity level by excluding the first 2 years of follow-up

(A) Alzheimer's disease

Alzheimer's disease

Physical activity level (MET-min/week)	Number of patients	Number of events	Incidence per 1,000 person-years	Adjusted HR* (95% CI)
Inactive (0)	21,826	586	19.9	1.00 (ref.)
Insufficiently active (1-499)	15,562	366	17.1	0.96 (0.84-1.09)
Active (500-999)	15,346	274	13.1	0.82 (0.71-0.95)
Highly active (≥1,000)	9,552	149	10.9	0.78 (0.64-0.93)

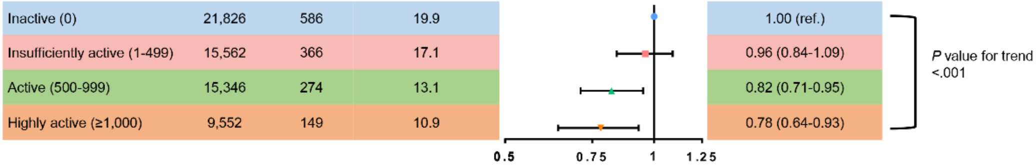

P value for trend <.001

Alzheimer's disease after censoring stroke

Physical activity level (MET-min/week)	Number of patients	Number of events	Incidence per 1,000 person-years	Adjusted HR* (95% CI)
Inactive (0)	21,826	579	19.8	1.00 (ref.)
Insufficiently active (1-499)	15,562	360	17.0	0.94 (0.83-1.08)
Active (500-999)	15,346	268	12.9	0.81 (0.70-0.93)
Highly active (≥1,000)	9,552	146	10.8	0.76 (0.63-0.92)

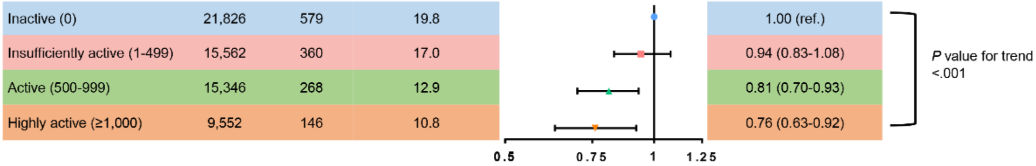

P value for trend <.001

(B) Vascular dementia

Vascular dementia

Physical activity level (MET-min/week)	Number of patients	Number of events	Incidence per 1,000 person-years	Adjusted HR* (95% CI)
Inactive (0)	21,826	101	3.3	1.00 (ref.)
Insufficiently active (1-499)	15,562	56	2.5	0.84 (0.60-1.16)
Active (500-999)	15,346	52	2.4	0.85 (0.60-1.20)
Highly active (≥1,000)	9,552	23	1.7	0.63 (0.40-1.01)

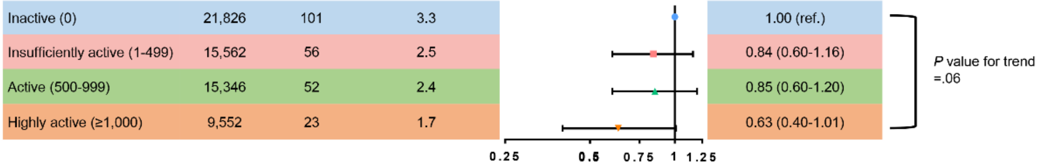

P value for trend =.06

Vascular dementia after censoring stroke

Physical activity level (MET-min/week)	Number of patients	Number of events	Incidence per 1,000 person-years	Adjusted HR* (95% CI)
Inactive (0)	21,826	97	3.2	1.00 (ref.)
Insufficiently active (1-499)	15,562	56	2.4	0.86 (0.62-1.20)
Active (500-999)	15,346	49	2.2	0.82 (0.58-1.17)
Highly active (≥1,000)	9,552	20	1.6	0.58 (0.39-1.00)

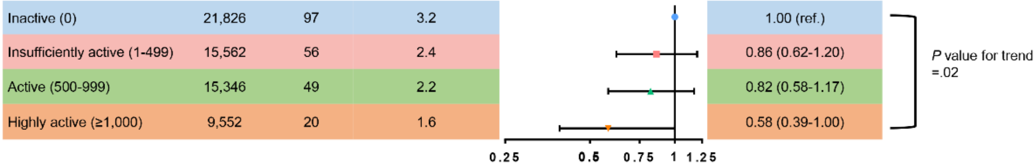

P value for trend =.02

(A) Alzheimer's disease, (B) Vascular dementia
Incident dementia occurring 2 years after enrollment was assessed to minimize reverse causation bias.
*The model was adjusted for age, sex, body mass index, Hospital Frailty Risk score, income, smoking, alcohol, hypertension, diabetes mellitus, dyslipidemia, chronic kidney disease, heart failure, vascular disease, prior ischemic stroke or transient ischemic attack, chronic obstructive pulmonary disease, and malignancy.
CI, confidence interval; HR, hazard ratio; MET, metabolic equivalent task.

© 2021 Yoon M et al. *JAMA Network Open*.

eFigure 4. Risk of dementia sub-type in relation to the total leisure-time physical activity level by including all follow-up period

(A) Alzheimer's disease

Alzheimer's disease

Physical activity level (MET-min/week)	Number of patients	Number of events	Incidence per 1,000 person-years	Adjusted HR* (95% CI)
Inactive (0)	21,826	1,211	17.0	1.00 (ref.)
Insufficiently active (1-499)	15,562	719	14.0	0.95 (0.87-1.04)
Active (500-999)	15,346	550	10.8	0.84 (0.76-0.93)
Highly active (≥1,000)	9,552	273	8.5	0.74 (0.65-0.85)

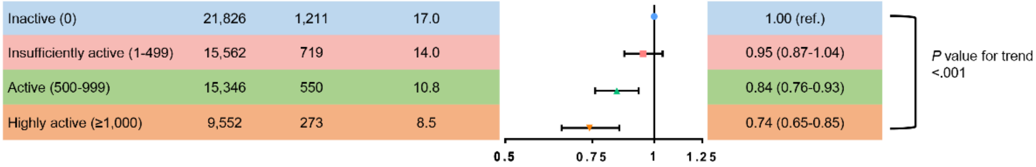

P value for trend <.001

Alzheimer's disease after censoring stroke

Physical activity level (MET-min/week)	Number of patients	Number of events	Incidence per 1,000 person-years	Adjusted HR* (95% CI)
Inactive (0)	21,826	1,171	16.8	1.00 (ref.)
Insufficiently active (1-499)	15,562	674	13.6	0.94 (0.86-1.04)
Active (500-999)	15,346	518	10.5	0.83 (0.75-0.92)
Highly active (≥1,000)	9,552	270	8.4	0.74 (0.65-0.85)

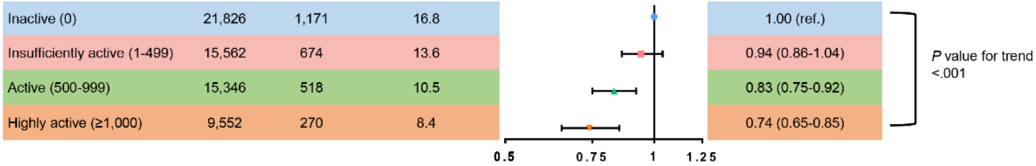

P value for trend <.001

(B) Vascular dementia

Vascular dementia

Physical activity level (MET-min/week)	Number of patients	Number of events	Incidence per 1,000 person-years	Adjusted HR* (95% CI)
Inactive (0)	21,826	208	2.9	1.00 (ref.)
Insufficiently active (1-499)	15,562	111	2.1	0.83 (0.66-1.05)
Active (500-999)	15,346	122	2.4	1.03 (0.82-1.29)
Highly active (≥1,000)	9,552	54	1.7	0.73 (0.53-1.00)

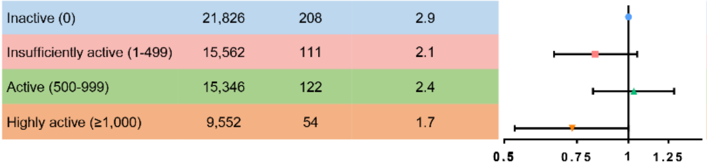

P value for trend =.11

Vascular dementia after censoring stroke

Physical activity level (MET-min/week)	Number of patients	Number of events	Incidence per 1,000 person-years	Adjusted HR* (95% CI)
Inactive (0)	21,826	175	2.5	1.00 (ref.)
Insufficiently active (1-499)	15,562	89	1.7	0.82 (0.63-1.06)
Active (500-999)	15,346	100	2.0	1.01 (0.78-1.30)
Highly active (≥1,000)	9,552	44	1.4	0.74 (0.52-1.04)

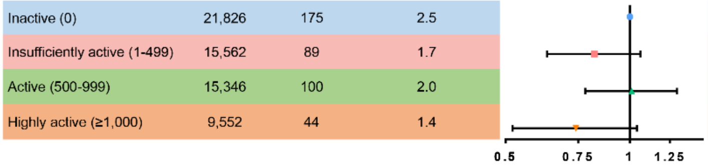

P value for trend =.24

(A) Alzheimer's disease, (B) Vascular dementia
*The model was adjusted for age, sex, body mass index, Hospital Frailty Risk score, income, smoking, alcohol, hypertension, diabetes mellitus, dyslipidemia, chronic kidney disease, heart failure, vascular disease, prior ischemic stroke or transient ischemic attack, chronic obstructive pulmonary disease, and malignancy.
CI, confidence interval; HR, hazard ratio; MET, metabolic equivalent task.

© 2021 Yoon M et al. *JAMA Network Open*.

eFigure 5. Cumulative incidence curve of Alzheimer's dementia according to leisure-time physical activity

(A) Excluding the first 2 years of follow-up

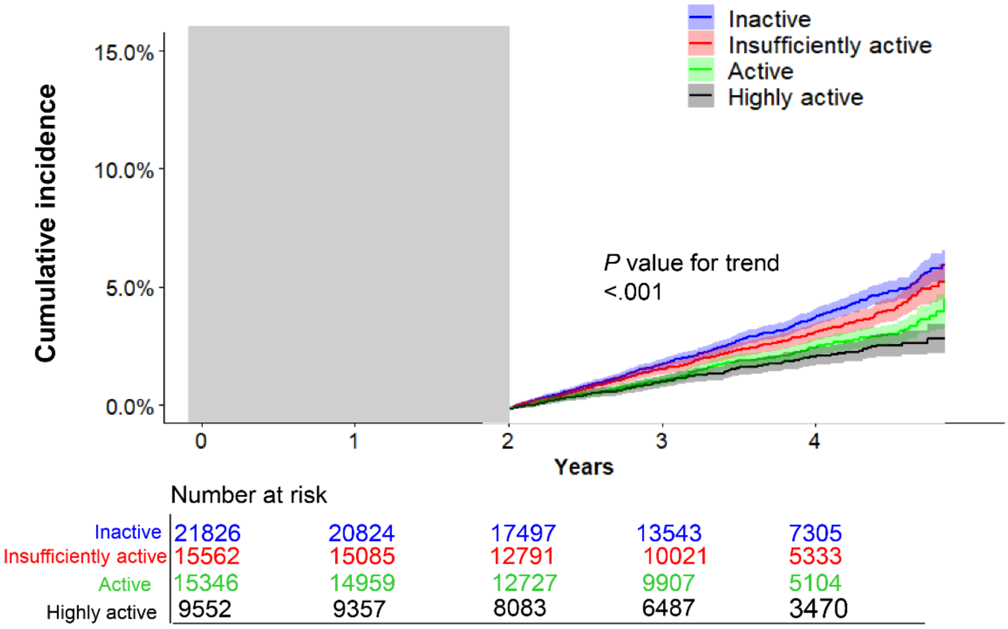

(B) Including all follow-up period

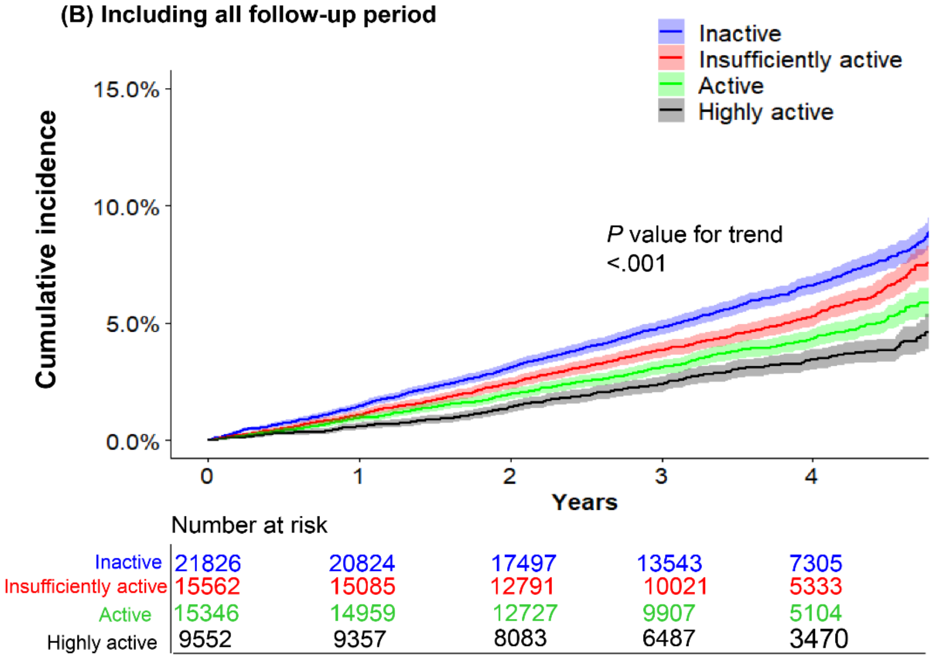

(A) Analyses by excluding the first 2 years of follow-up to minimize reverse causation bias. Incident dementia occurring 2 years after enrollment was assessed. (B) Analyses by including all follow-up period.

© 2021 Yoon M et al. *JAMA Network Open*.

eFigure 6. Cumulative incidence curve of vascular dementia according to leisure-time physical activity

(A) Excluding the first 2 years of follow-up

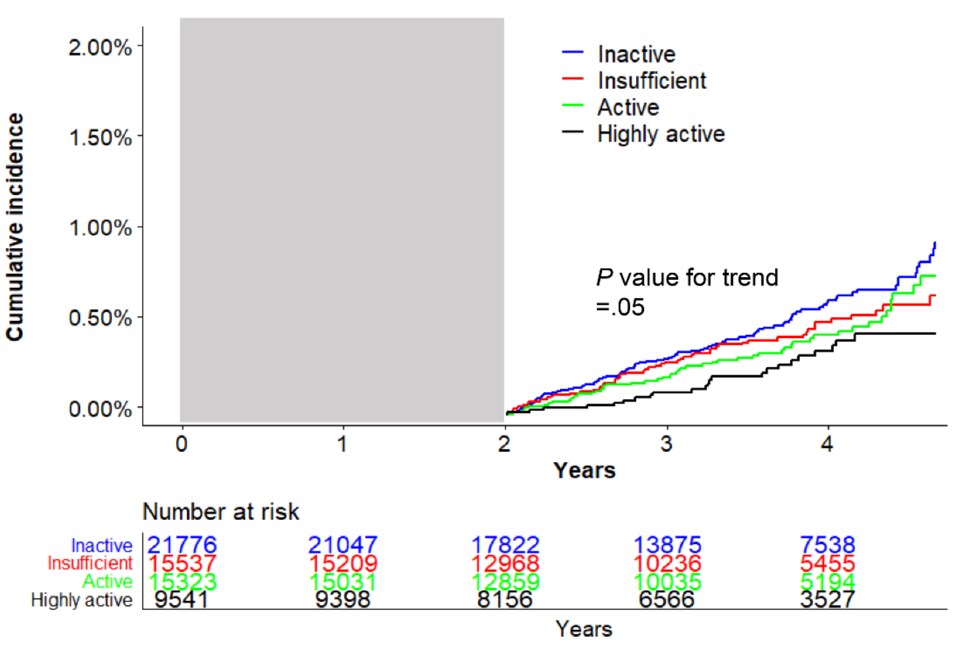

(B) Including all follow-up period

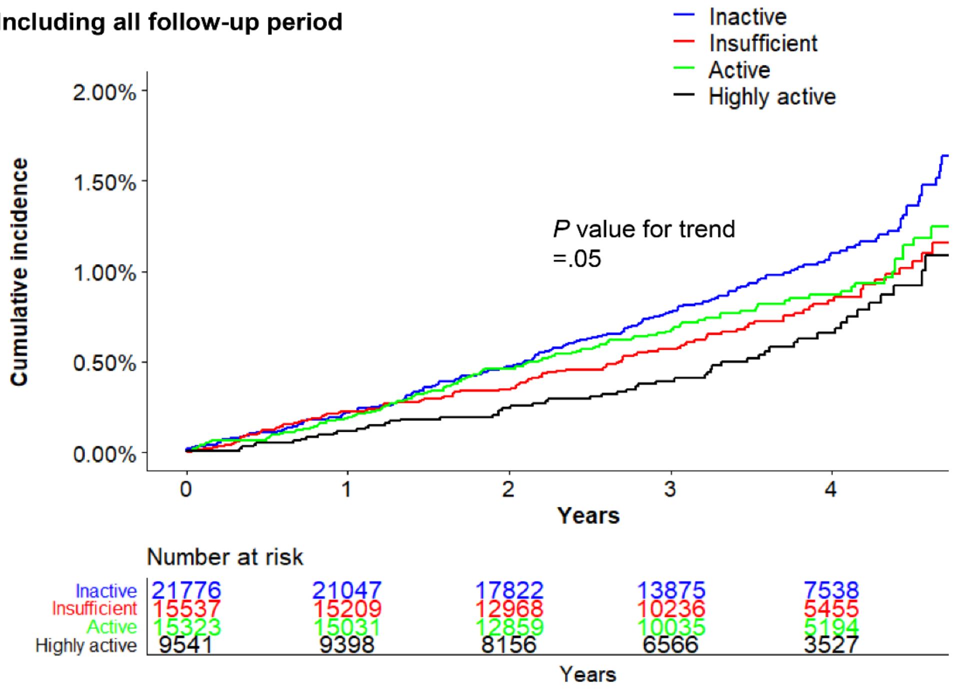

(A) Analyses by excluding the first 2 years of follow-up to minimize reverse causation bias. Incident dementia occurring 2 years after enrollment was assessed. (B) Analyses by including all follow-up period.

© 2021 Yoon M et al. *JAMA Network Open*.

eFigure 7. Risk of overall dementia in relation to the total leisure-time physical activity level by excluding participants with KDSQ score ≥4 (Total N = 60,066)

(A) Excluding the first 2 years of follow-up

Overall dementia

Physical activity level (MET-min/week)	Number of patients	Number of events	Incidence per 1,000 person-years	Fully adjusted HR* (95% CI)
Inactive (0)	21,151	768	21.2	1.00 (ref.)
Insufficiently active (1-499)	14,992	459	17.9	0.90 (0.80-1.00)
Active (500-999)	14,771	369	14.7	0.80 (0.71-0.92)
Highly active (≥1000)	9,152	182	11.5	0.71 (0.60-0.82)

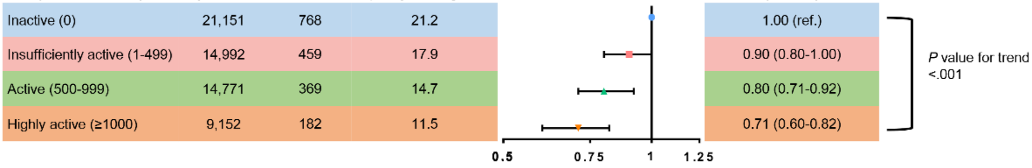

P value for trend <.001

Overall dementia after censoring stroke

Physical activity level (MET-min/week)	Number of patients	Number of events	Incidence per 1,000 person-years	Fully adjusted HR* (95% CI)
Inactive (0)	21,151	727	20.7	1.00 (ref.)
Insufficiently active (1-499)	14,992	428	17.6	0.90 (0.80-1.01)
Active (500-999)	14,771	341	14.3	0.80 (0.71-0.91)
Highly active (≥1000)	9,152	173	11.2	0.70 (0.60-0.80)

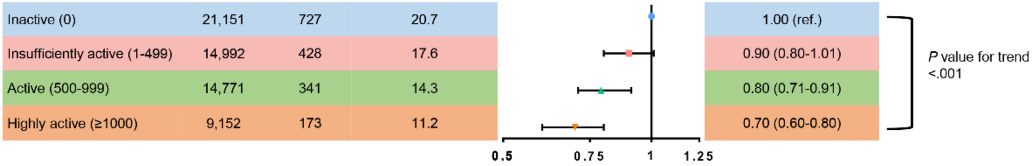

P value for trend <.001

(B) Including all follow-up period

Overall dementia

Physical activity level (MET-min/week)	Number of patients	Number of events	Incidence per 1,000 person-years	Adjusted HR* (95% CI)
Inactive (0)	21,151	1,626	23.8	1.00 (ref.)
Insufficiently active (1-499)	14,992	920	18.7	0.92 (0.84-0.99)
Active (500-999)	14,771	750	15.5	0.84 (0.77-0.92)
Highly active (≥1,000)	9,152	354	11.5	0.72 (0.64-0.81)

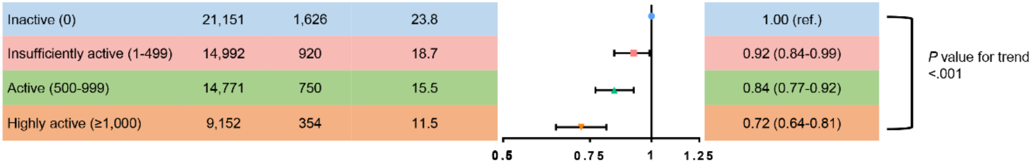

P value for trend <.001

Overall dementia after censoring stroke

Physical activity level (MET-min/week)	Number of patients	Number of events	Incidence per 1,000 person-years	Adjusted HR* (95% CI)
Inactive (None)	21,151	1,519	22.9	1.00 (ref.)
Insufficiently active (1-499)	14,992	852	17.8	0.91 (0.84-0.99)
Active (500-999)	14,771	688	14.6	0.83 (0.76-0.91)
Highly active (≥1,000)	9,152	325	10.8	0.71 (0.63-0.81)

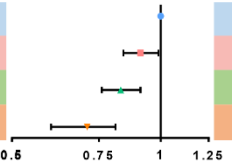

P value for trend <.001

Total KDSQ score ≥4 (N = 2,220) indicates possible cognitive impairment, which need further investigation to confirm cognitive dysfunction.
*The model was adjusted for age, sex, body mass index, Hospital Frailty Risk score, income, smoking, alcohol, hypertension, diabetes mellitus, dyslipidemia, chronic kidney disease, heart failure, vascular disease, prior ischemic stroke or transient ischemic attack, chronic obstructive pulmonary disease, and malignancy.
CI, confidence interval; HR, hazard ratio; KDSQ, Korean Dementia Screening Questionnaire; MET, metabolic equivalent task.

© 2021 Yoon M et al. *JAMA Network Open*.

eFigure 8. Risk of overall dementia in relation to the total leisure-time physical activity level by time-varying regression analyses

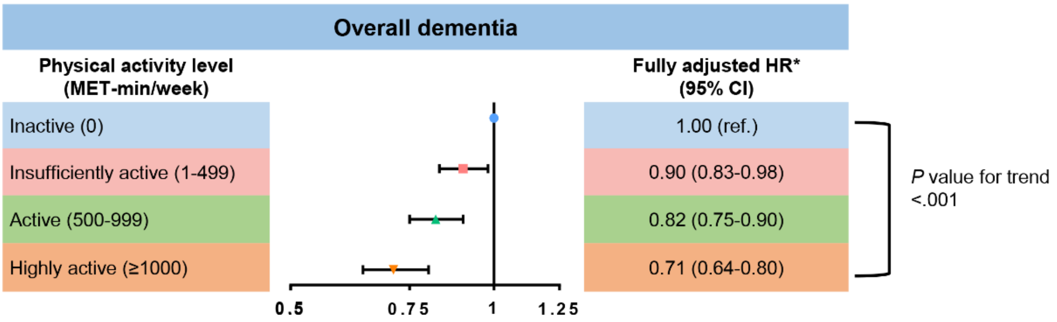

Physical activity levels were treated as a time-dependent variable.
*The model was adjusted for age, sex, body mass index, Hospital Frailty Risk score, income, smoking, alcohol, hypertension, diabetes mellitus, dyslipidemia, chronic kidney disease, heart failure, vascular disease, prior ischemic stroke or transient ischemic attack, chronic obstructive pulmonary disease, and malignancy.
CI, confidence interval; HR, hazard ratio; MET, metabolic equivalent task.

© 2021 Yoon M et al. *JAMA Network Open*.
